# A Correction

**Published:** 1905-11

**Authors:** 


A Correction. An article in "The Publisher's Department" in our September number on "Hemorrhage of the Bladder," in which Eusoma had been found effective, was, by a clerical error, credited to the *Medical World* instead of the *Medical Herald*.

